# Molecular typing and prevalence of antibiotic resistance and virulence genes in *Streptococcus agalactiae* isolated from Chinese dairy cows with clinical mastitis

**DOI:** 10.1371/journal.pone.0268262

**Published:** 2022-05-06

**Authors:** Guangli Han, Baohai Zhang, Zidan Luo, Biao Lu, Zhengzhong Luo, Jieru Zhang, Yin Wang, Yan Luo, Zexiao Yang, Liuhong Shen, Shumin Yu, Suizhong Cao, Xueping Yao

**Affiliations:** 1 College of Veterinary Medicine, Sichuan Agricultural University, Chengdu, Sichuan, China; 2 Key Laboratory of Animal Disease and Human Health of Sichuan Province, Chengdu, Sichuan, China; University of Illinois, UNITED STATES

## Abstract

Bovine mastitis is a common disease occurring in dairy farms and can be caused by more than 150 species of pathogenic bacteria. One of the most common causative organisms is *Streptococcus agalactiae*, which is also potentially harmful to humans and aquatic animals. At present, research on *S*. *agalactiae* in China is mostly concentrated in the northern region, with limited research in the southeastern and southwestern regions. In this study, a total of 313 clinical mastitis samples from large-scale dairy farms in five regions of Sichuan were collected for isolation of *S*. *agalactiae*. The epidemiological distribution of *S*. *agalactiae* was inferred by serotyping isolates with multiplex polymerase chain reaction. Susceptibility testing and drug resistance genes were detected to guide the clinical use of antibiotics. Virulence genes were also detected to deduce the pathogenicity of *S*. *agalactiae* in Sichuan Province. One hundred and five strains of *S*. *agalactiae* (33.6%) were isolated according to phenotypic features, biochemical characteristics, and 16S rRNA sequencing. Serotype multiplex polymerase chain reaction analysis showed that all isolates were of type Ia. The isolates were up to 100% sensitive to aminoglycosides (kanamycin, gentamicin, neomycin, and tobramycin), and the resistance rate to β-lactams (penicillin, amoxicillin, ceftazidime, and piperacillin) was up to 98.1%. The *TEM* gene (β-lactam-resistant) was detected in all isolates, which was in accordance with a drug-resistant phenotype. Analysis of virulence genes showed that all isolates harbored the *cfb*, *cylE*, *fbsA*, *fbsB*, *hylB*, and *α-enolase* genes and none harbored *bac* or *lmb*. These data could aid in the prevention and control of mastitis and improve our understanding of epidemiological trends in dairy cows infected with *S*. *agalactiae* in Sichuan Province.

## Introduction

*Streptococcus agalactiae*, also known as a group B *Streptococcus*, is a pathogen with high infectivity. The bacterium invades the mammary glands of dairy cows via the skin and teat and causes mastitis [1]. Mastitis caused by *S*. *agalactiae* is generally a chronic disease with few acute outbreaks and no significant clinical symptoms but reduces the milk yield and has severe economic consequences for dairy farms [2, 3]. The financial impact of mastitis includes the costs of treatment, milk that must be discarded, increased workload, reduced milk production, and culling and replacement [4]. Many types of *Streptococcus* cause bovine mastitis, the most important of which is *S*. *agalactiae* [5, 6]. *S*. *agalactiae* was under great control in northern Europe between the 1960s and 20^th^ century but became a re-emerging pathogen of dairy cattle and recognized as an emerging pathogen in human adults worldwide [7–10]. *S*. *agalactiae* was the causative organism in approximately 20%–40% of cases of bovine mastitis in China [11]. Moreover, *S*. *agalactiae* is known to cause serious infections in humans, including infant sepsis, endocarditis, meningitis, and pneumonia in newborns, the elderly, and pregnant women [12–17]. It can also infect aquatic animals [18, 19].

In cows, the main route of entry for *S*. *agalactiae* is via the teat, but infection can also occur via the oral–fecal route and directly or indirectly trigger mastitis [1, 20]. The pathogenicity of a bacterium depends on multiple virulence factors, which in the case of *S*. *agalactiae* include strong adsorption and anti-phagocytosis and immune evasion mechanisms [21]. A variety of surface proteins and endotoxins, including hemolysins and the Christie–Atkins–Munch–Peterson factor, can increase the ability of *S*. *agalactiae* to invade and colonize its host. Furthermore, certain pathogenic factors, including fibrinogen binding (*fbs A/B*) proteins, adhesion (*lmb*) proteins, and enolase proteins, can damage tissues in the host and cause destruction in the immune system. These virulence factors promote survival and spread of bacteria and seriously compromise the health of both animals and humans [22, 23]. One of the important virulence factors in *S*. *agalactiae* is capsular polysaccharide, which has characteristic antigenicity features and differential properties that are useful for serotyping. The studies reported so far have shown that *S*. *agalactiae* can be divided into 10 types (Ia, Ib, II–IX) according to the structure of its capsular polysaccharide [24].

Bovine mastitis is a major problem in the dairy industry, costing billions of dollars every year throughout the world, including in China, and *S*. *agalactiae* is one of the most important causative pathogens. At present, antibiotics are the first-line treatment for bovine mastitis. However, with the growing issue of antibiotic resistance and emergence of resistant organisms, antibiotics are becoming ineffective. Moreover, there is the problem of antibiotic residues, which are a danger to public health [25, 26]. Researchers have found an association between antibiotic resistance in *S*. *agalactiae* and resistance genes within the organism, which can transfer with migration of drug-resistant bacteria to originally drug-susceptible bacteria, which then also become drug-resistant [27]. The increase in drug-resistant strains has led to further increases in the use of antibiotics, which will not only lead to environmental pollution but also threaten human health. Therefore, the purpose of this study was to provide basic data for prevention and control of bovine mastitis by investigating *S*. *agalactiae* infection in dairy cows in Sichuan Province, determining drug resistance and carriage of resistance genes in isolated strains, and describing the distribution of virulence genes in isolates.

## Materials and methods

### Animal welfare statement

This study was carried out in strict accordance with the recommendations in the guide for the care and use of laboratory animals and approved by the Committee on Experimental Animal Management of the Sichuan Agricultural University. All farm owners in this study verbally agreed with the collection of milk samples.

### Collection of milk samples and isolation of bacteria

**Fig 1:**
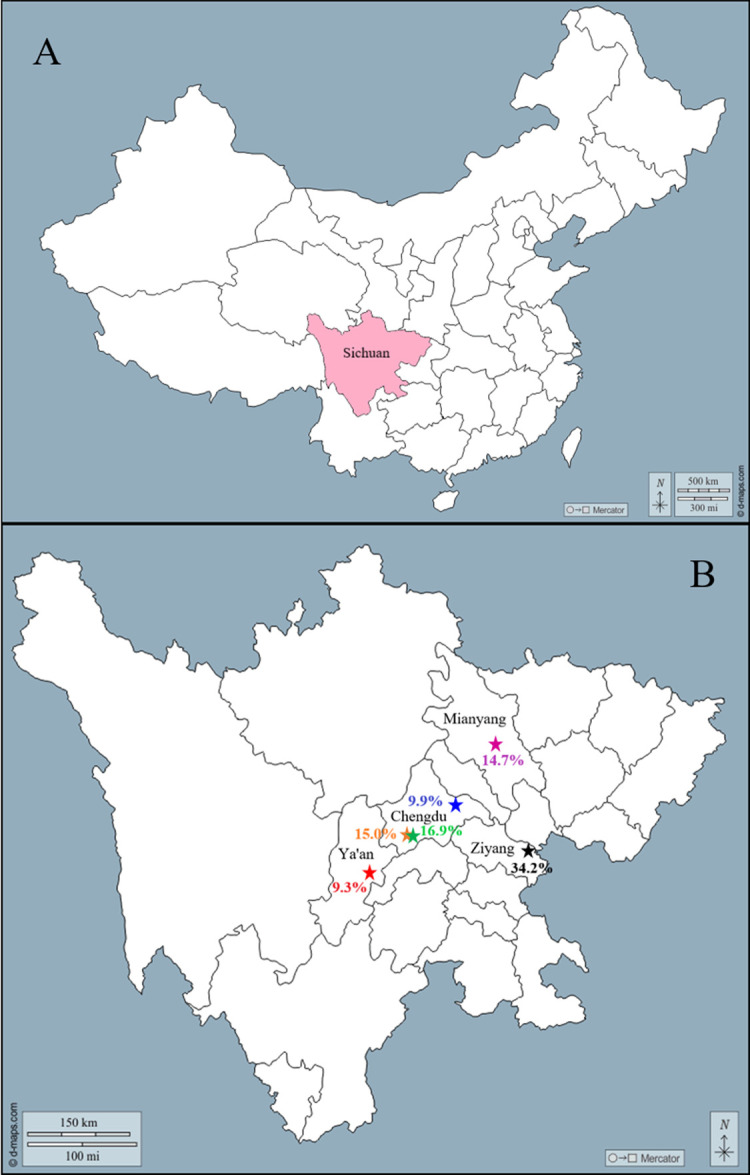
Geographical location of sampling. A, map of China, the colored plate represents Sichuan Province; B, map of Sichuan Province, red star represents Hongya farm, orange star represents Yangba farm, green star represents Yushu farm, blue star represents Qingbaijiang farm, purple star represents Songya farm, and black star represents Ninggang farm, percentages identical to the symbol color indicate the sample collection rate in that dairy farm. All original maps were download from d-maps.com.

Visible inflammation of the mammary glands and milk degeneration can be used to for comprehensive diagnosis of clinical-type mastitis [28]. A total of 313 milk samples were collected from cows with clinical mastitis from dairy farms in several regions of Sichuan Province between 2017 and 2019, specific sampling times, sampling rates and geographic locations are detailed in Table 1 and Fig 1. During the course of the study, we maintained cooperative relationships with six dairy farms in several region of Sichuan, China. When the cattle showed clinical symptoms, the dairy farmers would notify us to come collect samples and perform pathogen detection. To this end, we had prepared aseptic centrifuge tubes to collect quarter-level milk; these were placed in a foam box filled with ice bags and transported to the laboratory. All samples were obtained aseptically and sent to the laboratory within 12 hours, where they were inoculated on basic culture medium of Columbia agar containing 5% defibrinated sheep blood at 37°C for 18–24 h.

**Table 1:** Geographic distribution of samples collected in this study.

Region	Name of dairy farm	Isolates, n				
		2017 Sep–Dec	2018 Mar–Jun	2018 Sep–Dec	2019 Mar–Jun	Total
Qionglai	Yangba^a^	-	7	20	20	47
	Yushu^b^	-	28	18	7	53
Anyue	Ninggang^c^	25	33	35	14	107
Mianyang	Songya^d^	10	15	12	9	46
Qingbaijiang	Qingbaijiang^e^	-	6	5	20	31
Hongya	Hongya^f^	-	6	8	15	29
Total		35	95	98	85	313

a and b, belong to Youran Dairy Co., Ltd.

c, belongs to Ninggang Dairy Co., Ltd.

d, belongs to Sichuan Xuebao Dairy Group Co., Ltd.

e, belongs to Sichuan New Hope West China Animal Husbandry Co., Ltd.

f, belongs to Modern Farming Co., Ltd.

### Biochemical characterization

A single clone of each isolated strain was stained, and the suspected positive *Streptococcus spp*. (purple, spherical, and chain like) was tested with catalase and the Christie–Atkins–Munch–Peterson (CAMP) assay [25]. The existence of bubbles indicates catalase positivity after treating bacteria with 3% hydrogen peroxide [29]. The CAMP test was assessed with *Staphylococcus aureus*, CAMP positivity was indicated by significant hemolysis between the vertical but not the intersecting two bacteria after incubation for 18–24 h at 37°C. After Gram staining and biochemical testing, the isolates that were Gram positive, catalase-negative, and CAMP-positive were confirmed with 16S rRNA polymerase chain reaction (PCR).

### Genomic DNA extraction and 16S rRNA sequence analysis

Genomic DNA was extracted from bacteria cultured (incubated in brain heart infusion broth at 37°C overnight) using a bacterial DNA extraction kit (TIANamp Bacteria DNA Kit, TIANGEN BIOTECH (BEIJING) CO., LTD) according to the manufacturer’s instructions. The extracted DNA was stored at -20°C for future use.

DNA samples were determined with partial 16S rRNA sequencing, whereby forward (5’- AGAGTTTGATCCTGGCTCAG -3’) and reverse (5’- GGTTACCTTGTTACGACTT -3’) primers were used to amplify a product of approximately 1500 bp [30]. The amplified products were sent to Tsingke Biotechnology Co., Ltd. (Beijing, China) for Sanger sequencing, and the sequences were compared against those in the nucleotide database at the National Center for Biotechnology Information.

### Serotyping

Multiplex PCR was used for detection of serotypes using the method described by Imperi et al. [31] All primers were used at a concentration of 250 nM except for primers cpsI-Ia-6-7-F and cpsI-7-9-F, for which the concentration was 400 nM. The reaction mixture (25 μL) was amplified with an initial denaturation step at 95°C for 5 min, followed by 15 cycles of denaturation at 95°C for 1 min, annealing at 54°C for 1 min, and extension at 72°C for 2 min, followed by 25 cycles of denaturation at 95°C for 1 min, annealing at 56°C for 1 min, and extension at 72°C for 2 min, with a final extension at 72°C for 10 min.

### Analysis of antimicrobial susceptibility

All *S*. *agalactiae* isolates were tested for susceptibility to 10 antimicrobial agents that were frequently used in local dairy farms, including piperacillin (100 μg), ceftriaxone (30 μg), penicillin (10 U), amoxicillin (20 μg), ceftazidime (30 μg), kanamycin (30 μg), gentamicin (10 μg), neomycin (30 μg), streptomycin (10 μg), and tobramycin (10 μg) using the disc diffusion method on Mueller-Hinton agar plates. The cultures were incubated overnight at 37°C, and the results were interpreted in accordance with the recommendations of the Clinical and Laboratory Standards Institute (formerly the National Committee for Clinical Laboratory Standards 2020).

### Detection of resistance and virulence genes

All *S*. *agalactiae* isolates were screened for the presence of the following resistance genes: *TEM*, *IMP*, *DHA*, and *OXA* (β-lactam resistance genes) and *aph(3’)Ia*, *ant(3’)I*, *aac(6’)Ib*, and *aac(3’)Ib* (aminoglycoside resistance genes). PCR was performed using specific primers, the amplification conditions for which are shown in Table 2. A total of 20 μL of reaction mixture was prepared with 10 μL of 2×*Taq* Master Mix (Beijing Solarbio Science & Technology Co., Ltd), 1 μL of template DNA, 0.5 μL of each primer (10 μM), and 8 μL of distilled water. Initial denaturation at 94°C for 3 min was followed by 34 cycles of amplification at 94°C for 20 s, annealing at specific temperatures (Table 2) for 20 s, extension at 72°C for 45 s, and a final extension step at 72°C for 5 min. The amplified PCR products were visualized on 1% agarose gel using a gel documentation system (GeneGenius Bio Imaging System; Syngene, Bangalore, India).

**Table 2:** Primers of resistance and virulence genes.

Gene	Sequence (5’ to 3’)	Annealing temperature (°C)	Amplicon size (bp)	Reference
**Resistance gene**				
*TEM*	F: CATTTCCGTGTCGCCCTTAT R: GACCGAGTTGCTCTTGCC	55	259	[32]
*OXA*	F: AGCAGCGCCAGTGCATCA R: ATTCGACCCCAAGTTTCC	58	587	[32]
*IMP*	F: CGGCCTCAGGAGACGGCTTT R: AACCAGTTTTGCCTTACCAT	56	405	[33]
*DHA*	F: AACTTTCACAGGTGTGCTGGGT R: CCGTACGCATACTGGCTTAGC	58	708	[34]
*aph(3’)Ia*	F: TGACTGGGCACAACAGACAA R: CGGCGATACCGTAAAGCAC	58	677	[35]
*ant(3’)I*	F: TGATTTGCTGGTTACGGTGAC R: CGCTATGTTCTCTTGCTTTTG	56	284	[36]
*aac(6’)Ib*	F: ATGACCTTGCCATGCTCCTATGA R: CGAATGCCTGGCGTGTTT	58	486	[37]
*aac(3’)Ib*	F: ACCCTACGAGGAGACTCTGAATG R: CCAAGCATCGGCATCTCATA	55	384	[37]
**Virulence gene**				
*bac*	F: AAGGCTATGAGTGAGAGCTTGGAG R: CTGCTCTGGTGTTTTAGGAACTTG	55	604	[38]
*cfb*	F: AAGCGTGTATTCCAGATTTCC R: AGACTTCATTGCGTGCCAAC	56	317	[39]
*cylE*	F: CATTGCGTAGTCACCTCCC R: GGGTTTCCACAGTTGCTTGA	56	380	[40]
*fbsA*	F: GAACCTTCTTGTCACACTTG R: TTGATCCTAGCACTCCCA	58	556	[40]
*fbsB*	F: GCGCAAACTTCTGTCCAA R: CCGATACGATTGTCCAAATG	58	417	[40]
*hylB*	F: CACCAATCCCCACTCTACTA R: TGTGTCAAACCATCTATCAG	56	444	[41]
*α-enolase*	F: ATGTCAATTATTACTGATGTTTACGC R: CTATTTTTTTAAGTTGTAGAATGATT	55	1038	[42]
*lmb*	F: CCGTCTGTAAATGATGTGGC R: GAAATACCCGAGATACCAAG	55	473	[41]

F, forward; R, reverse

The virulence genes screened were based on those found in humans and were as follows: *bac* (C-β protein), *cfb* (CAMP factor), *cylE* (β-hemolysins/cytolysin), *fbsA* (the fibrinogen-binding protein FbsA), *fbsB* (the fibrinogen-binding protein FbsB), *hylB* (hyaluronate lyase), *α-enolase*, and *lmb* (laminin-binding protein) [19]. The content of the reaction mixture and the amplification program were the same as those described above for the detection of resistance genes.

## Results

### Isolation and identification of *S*. *agalactiae*

After Gram staining, biochemical analysis, and 16S sequencing analysis, 105 bacterial isolates in 313 milk samples were identified to be *S*. *agalactiae*, with an isolation rate of 33.6%.

### Serotyping of *S*. *agalactiae*

Multiplex PCR detection was performed to differentiate the 10 capsular serotypes of *S*. *agalactiae* (Ia, Ib, II–IX). Two bands of 688 bp and 272 bp appeared in all 105 isolated strains, including that all 105 isolated *S*. *agalactiae* serotypes were of type Ia.

### Antimicrobial susceptibility

**Fig 2:**
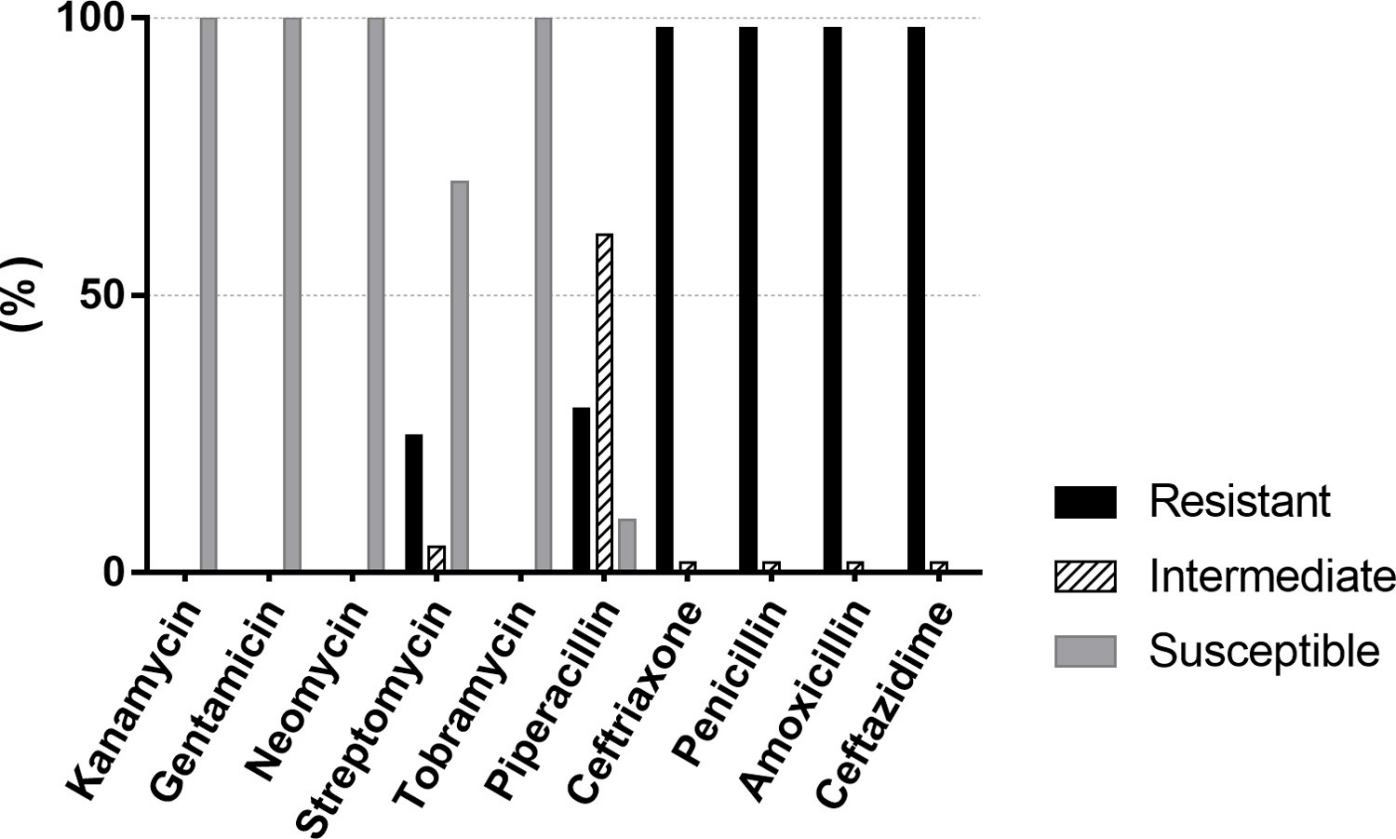
Antibiotic susceptibility profiles of 105 isolates. The first five drugs on the x-axis are aminoglycosides and the last five are β-lactams.

The strains were judged to be susceptible, intermediate, or resistant to the different antibiotics according to the regulations of the executive standard of antimicrobial susceptibility testing issued by the Clinical and Laboratory Standards Institute. Drug susceptibility testing of the isolates showed that up to 100% of isolates were susceptible to aminoglycosides (kanamycin, gentamicin, neomycin, and tobramycin) and that 70.5% were susceptible to streptomycin. All 105 isolates were resistant to the β-lactam agents (penicillin, amoxicillin, ceftazidime, and ceftriaxone), with a resistance rate of up to 98.1%. The resistance rate for piperacillin was 29.5% (Fig 2; the original data are shown in S1 Table, and the breakpoints for each antibiotic are shown in S2 Table).

### Prevalence of antimicrobial resistance and virulence genes

PCR analysis was used to determine the drug resistance and virulence gene profiles of the *S*. *agalactiae* isolates. The frequencies of these genes in the 105 isolates are shown in S3 Table. Eight resistance genes for β-lactams (*TEM*, *IMP*, *DHA*, *OXA*) and aminoglycosides (*aph(3’)Ia*, *ant(3’)I*, *aac(6’)Ib*, *aac(3’)Ib*) were examined in 105 *S*. *agalactiae* isolates. Only *TEM* genes for β-lactams were detected in the isolated strains; the detection rate was up to 98%, and other resistance genes were not detected.

Eight virulence genes, namely *lmb*, *cylE*, *α-enolase*, *fbsA*, *fbsB*, *cfb*, *hylB*, and *bac*, were targeted for detection. The results showed 100% detection of *cfb*, *cylE*, *fbsA*, *fbsB*, *hylB*, and *α-enolase* for all 105 *S*. *agalactiae* isolates; the *bac* and *lmb* genes were not detected in these isolates.

## Discussion

Bovine mastitis has a complex etiology and can be caused by a variety of pathogenic microorganisms, including bacteria, viruses, and fungi. However, many studies have shown that bacteria are still the main causative pathogens for bovine mastitis and that *S*. *agalactiae* is one of the most important [43, 44]. In this study, 313 milk samples from dairy cows with clinical mastitis were collected from several areas in Sichuan Province. A total of 105 strains of *S*. *agalactiae* were isolated from these samples, for an isolation rate of 33.6%. Our microbiological data are comparable with those reported by Zeryehun et al. (21.2%) in Ethiopia [45]. However, our isolation rates were significantly higher than those reported by Chehabi et al. (4.3%) in Denmark and Tomazi et al. (5.9%) in Brazil [46, 47].

The isolation rate for *S*. *agalactiae* in clinical mastitis samples varies from region to region according to the local climate and breeding environment. Even in the same country, diverse prevalence rate can be found across regions. In our study, all 105 strains were of capsular type Ia. In a study by Wang et al., serotype II of *S*. *agalactiae* was the most prevalent in dairy cows in Jiangsu, China, whereas the capsular serotypes isolated from neonates and pregnant women by Rogers et al. were mainly of type III and those in our study in dairy cows were of type Ia; we consider that although serotype II was found to be more prevalent in Jiangsu, China, serotype Ia is the most common type in cattle [10, 20, 48–52]. These inconsistent findings may reflect geographic and host differences in these isolates. The collected data on the distribution of serotypes in different geographical regions should serve as a basis for the development of vaccine proposals [53, 54]. Our findings were based on *S*. *agalactiae* isolates from milk samples collected in Sichuan Province and may reflect the infection status of dairy cows with mastitis throughout southwest China; however, our data cannot be considered representative of all Chinese provinces. This is a weakness of the present study. However, along with the wide study of bovine mastitis in northern China, our study could supplement and perfect the panorama of pathogens causing clinical mastitis in China dairy herds, although the samples were not geographically evenly distributed across the country. At the nationwide level, the *S*. *agalactiae* isolation rate is significantly higher in the south than in the north, indicating that prevention and control of this bacterium remains challenging in the southern regions.

Although vaccines against the common pathogens that cause bovine mastitis are available for prevention and control purposes, systemic or intramammary antibiotic therapy continues to be the mainstay of treatment for clinical mastitis [55]. Penicillin was used as the first choice for prevention and therapy of group B streptococcal (GBS); however, increased resistance of GBS to penicillin has been periodically reported since 1994 [56–59]. Streptococci have been shown to be highly resistant to enrofloxacin, erythromycin, lincomycin, and penicillin [60]. In this study, we performed susceptibility testing for 10 antibiotic agents (including β-lactams and aminoglycosides) commonly used to treat clinical mastitis in dairy cows located in Sichuan Province, and found that the resistance rate in 105 isolates was up to 98.1% for β-lactams (penicillin, amoxicillin, ceftriaxone, and ceftazidime) while all isolates possessed high sensitivity to aminoglycosides (kanamycin, gentamicin, neomycin, and tobramycin). These findings are consistent with those of a study performed in Ukraine by Elias et al., who isolated *S*. *agalactiae* that possessed high resistance to β-lactam antibiotics, and those of a study in North China by Tian et al., who isolated streptococci with a resistance rate of 100% to penicillin and average sensitivity to aminoglycosides (92.86%) [61, 62]. These susceptibility results correspond to the clinical use of antibiotics in China. However, in a study performed in Slovakia, 23 *S*. *agalactiae* strains showed resistance rates of 8.7% and 30.4% to oxacillin and streptomycin, respectively, but were highly susceptible to penicillin and ceftiofur [63]. Furthermore, all streptococci in Denmark were found to be susceptible to penicillin [46]. All isolates were resistant to aminoglycosides while sensitive to β-lactam antibiotics and rifampicin in the Emilia Romagna region (Northern Italy) [64]. The inconsistency between these reports and our present findings may reflect differences in the types of antibiotics used in clinical practice across regions. The main antibiotics used to treat bovine mastitis in China are the β-lactams, although aminoglycosides may be used in the future to treat mastitis caused by *Streptococcus spp*.

PCR detection of resistance genes identified the *TEM* gene for β-lactams in all isolates; however, no other drug resistance genes were detected. In a study by Lu et al., the detection rate for genes conferring resistance to β-lactams was 100% in 10 strains of *S*. *agalactiae* resistant to penicillin [65]. Meanwhile, Yang et al. reported the detection rates for four aminoglycoside resistance genes [*aph (3’)-Ia*, *ant (3’)-I*, *aac (3’)-Ib* and *aac (6’)-Ib*] to be 0.0%, 75.0%, 0.0%, and 31.3%, respectively; overall, these values were consistent with the results of our study [66]. The combination of the data regarding the resistance genes and phenotypes indicates high resistance of *S*. *agalactiae* to β-lactams, but sensitivity to aminoglycosides, in Sichuan. Therefore, aminoglycosides may become the preferred agents for treatment of dairy cows with clinical mastitis in the future.

In this study, PCR showed that the *cfb*, *cylE*, *fbsA*, *fbsB*, *hylB*, and *enolase* virulence genes were present in all isolates, whereas the *bac* and *lmb* genes were not; the detection rates were the same with those reported for isolates in Argentina [67]. These virulence factors provide essential assistance for allowing pathogenic bacteria to invade the host and to be directly involved in the invasion process [68]. Notably, *cylE* is a pore-forming toxin involved in tissue damage and systemic dissemination of bacteria [69]. Previous studies have reported the presence of this gene in 78% of Polish strains and in 100% of Chinese strains [70, 71]. *FbsA* and *fbsB* were mainly present in type Ia and type III GBS, both major capsular types that could induce mastitis [5]. Moreover, *FbsA* and *fbsB* were prove to be closely associated with the adhesion of virulence factors [72]. *HylB* is regarded a dominant virulence factor in *S*. *agalactiae*, and the presence of *hylB* could enhance virulence when there is *S*. *agalactiae* mammary-gland invasion [73]. *Cfb* was widely detected in *S*. *agalactiae*; the CAMP factor produced by gene *cfb* could enhance the dissolution of sheep erythrocytes by *S*. *aureus*, leading to a CAMP-positive phenomenon as we found in this study [74]. Earlier molecular reports showed that in contrast to human isolates, most bovine isolates lack surface protein-encoding genes, including *lmb*, in line with our findings [75].

Our results could lay the foundation for mastitis prevention and control, selection of antimicrobial agents, and research regarding the mechanisms of bacterial infection in dairy cows in Sichuan Province. Our data suggest that aminoglycosides could be used to treat clinical mastitis caused by *S*. *agalactiae* in Sichuan Province. As a temporary measure, aminoglycosides could be useful in terms of clinical treatment and reduction of economic loss. However, *S*. *agalactiae* may eventually develop resistance to these antimicrobial agents and even transfer this ability to other bacteria with initial sensitivity. Therefore, use of aminoglycosides cannot be considered a long-term solution, and it is necessary to continue the search for alternative agents, such as vaccines and phage therapy. Moreover, the virulence factors detected in this study could provide basic data for vaccine preparation in southern regions, given reduced use of antibiotics and lesser efficiency of the GBS vaccines that have been produced.

### Statistical analysis

The data are presented as counts and percents. The image of the susceptibility assay was generated with GraphPad Prism version 7 (GraphPad Software, San Diego, CA).

## Supporting information

S1 Table Antibiotic susceptibility profiles of 105 *S*. *agalactiae* isolates from dairy cows. (PDF)

S2 Table Breakpoints for each antibiotic used in this antimicrobial susceptibility test. (PDF)

S3 Table Frequency of antibiotic resistance and virulence genes in the 105 *S*. *agalactiae* isolates. (PDF)
