# Supplementary material for: Molecular typing and prevalence of antibiotic resistance and virulence genes in Streptococcus agalactiae isolated from Chinese dairy cows with clinical mastitis
Source: PLoS One. 2022 May 6;17(5):e0268262. doi: 10.1371/journal.pone.0268262 (PMC9075616; doi:10.1371/journal.pone.0268262)
Supplement: S1 Table — (PDF) [file pone.0268262.s001.pdf]

S 1 Table. Antibiotic susceptibility profiles of 105 *S. agalactiae* isolates from dairy cows.

Antibiotic	Resistant no.	Intermediate no.	Susceptible no.
Kanamycin (30 μg)	0 (0%)	0 (0%)	105/105 (100%)
Gentamicin (10 μg)	0 (0%)	0 (0%)	105/105 (100%)
Neomycin (30 μg)	0 (0%)	0 (0%)	105/105 (100%)
Streptomycin (10 μg)	26/105 (24.8%)	5/105 (4.8%)	74/105 (70.5%)
Tobramycin (10 μg)	0 (0%)	0 (0%)	105/105 (100%)
Piperacillin (100 μg)	31/105 (29.5%)	64/105 (61.0%)	10/105 (9.5%)
Ceftriaxone (30 μg)	103/105 (98.1%)	2/105 (1.9%)	0 (0%)
penicillin (10 units)	103/105 (98.1%)	2/105 (1.9%)	0 (0%)
Amoxicillin (20 μg)	103/105 (98.1%)	2/105 (1.9%)	0 (0%)
Ceftazidime (30 μg)	103/105 (98.1%)	2/105 (1.9%)	0 (0%)
