# Supplementary material for: Molecular typing and prevalence of antibiotic resistance and virulence genes in Streptococcus agalactiae isolated from Chinese dairy cows with clinical mastitis
Source: PLoS One. 2022 May 6;17(5):e0268262. doi: 10.1371/journal.pone.0268262 (PMC9075616; doi:10.1371/journal.pone.0268262)
Supplement: S2 Table — (PDF) [file pone.0268262.s002.pdf]

S 2 Table. Breakpoints for each antibiotic used in this antimicrobial susceptibility test.

Antibiotic	Diameter of inhibition zone (mm)		
	Resistant	Intermediate	Susceptible
Kanamycin (30 μg)	≤13	14-17	≥18
Gentamicin (10 μg)	≤12	13-14	≥15
Neomycin (30 μg)	≤12	13-16	≥17
Streptomycin (10 μg)	≤11	12-14	≥15
Tobramycin (10 μg)	≤12	13-14	≥15
Piperacillin (100 μg)	≤17	18-20	≥21
Ceftriaxone (30 μg)	≤14	15-17	≥18
penicillin (10 units)	≤19	20-27	≥28
Amoxicillin (20 μg)	≤13	14-17	≥18
Ceftazidime (30 μg)	≤14	15-17	≥18
