# Supplementary material for: Molecular typing and prevalence of antibiotic resistance and virulence genes in Streptococcus agalactiae isolated from Chinese dairy cows with clinical mastitis
Source: PLoS One. 2022 May 6;17(5):e0268262. doi: 10.1371/journal.pone.0268262 (PMC9075616; doi:10.1371/journal.pone.0268262)
Supplement: S3 Table — (PDF) [file pone.0268262.s003.pdf]

S 3 Table. Frequency of antibiotic resistance and virulence genes in the 105 *S. agalactiae* isolates.

Antibiotic Resistance Genes	Frequency (%)	Virulence Genes	Frequency (%)
TEM	98	*cfb*	100
IMP	0	*cylE*	100
DHA	0	*fbsA*	100
OXA	0	*fbsB*	100
aph(3')Ia	0	*hylB*	100
ant(3')I	0	*α-enolase*	100
aac(6')Ib	0	*bac*	0
aac(3')Ib	0	*lmb*	0
